# Molecular Mechanisms of Symptom Fluctuations in Malignant Tumor Patients: Stable by Day and Severe at Night

**DOI:** 10.7150/jca.128423

**Published:** 2026-02-18

**Authors:** Zhongxuan Xie, Wei Jin, Juezhou Pang, Kangshuo Hu, Lihong Li

**Affiliations:** 1 The Second Clinical Medical College of Zhejiang Chinese Medical University, Hangzhou, Zhejiang Province 310000, PR China.; 2 School of Traditional Chinese Medicine, Beijing University of Chinese Medicine, Beijing 100029, PR China.; 3 The Second Affiliated Hospital of Zhejiang Chinese Medical University, Hangzhou, Zhejiang Province 310005, PR China.

**Keywords:** tumor symptoms, circadian rhythm, biological clock, molecular mechanisms, immune rhythm

## Abstract

Patients with malignant tumors often experience fluctuations in the severity of their symptoms depending on the time of day. In traditional Chinese medicine, symptoms are said to follow a pattern of "mild in the morning, stable by day, worsening in the evening, and severe at night." This article investigates the circadian chronobiology of symptoms and examines their molecular pathophysiology. Evidence suggests that disruptions in core circadian clock genes, such as BMAL1 and PER, along with the dysregulation of cellular metabolic pathways, immune responses, and endocrine functions, synergistically facilitate tumor growth and metastasis during nocturnal periods. These molecular alterations contribute to symptom exacerbation through mechanisms which include direct tumor invasion, neural infiltration, inflammatory processes, dorsal root ganglion (DRG) sensitization, and abnormal melatonin secretion. The article further explores three chronotherapeutic strategies and assesses melatonin's role in targeted oncological therapy, aiming to optimize circadian regulation and symptom management, thereby providing a scientific foundation for personalized anti-tumor interventions that are based on circadian rhythms.

## Introduction

In this age of rapid economic development, environmental pollution, ageing societies and increasing stress, the spectrum of human diseases has changed significantly. Malignant tumors have become a predominant global public health challenge, societal and economic burden in the 21st century, seriously threatening human health and hindering the extension of life expectancy. It is predicted that malignant tumors will replace cardiovascular diseases and infectious diseases as the leading cause of death in most countries by the end of this century^1^. In the United States, projections estimate approximately two million new cancer diagnoses and up to 610,000 cancer-related deaths by 2025^2^. In China, the incidence of malignant tumors similarly continues to rise^3^. Notably, symptoms in cancer patients often exhibit distinct diurnal fluctuations. In traditional Chinese medicine, symptoms are described as typically “mild in the morning, stable by day, worsening in the evening, and severe at night.” Especially common among patients in advanced stages of illness are symptoms such as pain, sleep disorder, nausea, vomiting, anaemia, and fatigue^4^, ^5^ - all symptoms which exhibit this circadian rhythm. Diurnal fluctuations in symptoms not only exacerbate patient suffering but also significantly increase the burden on caregivers, family members, and healthcare teams.

The circadian clock is a collection of endogenous cellular oscillators with a 24-hour cycle present in the majority of cells in the body. It precisely regulates physiological processes and is crucial for maintaining homeostasis^6^. Disruption of the circadian clock can directly interfere with circadian gene expression, leading to disruptions of the cell cycle and genomic instability, thereby increasing the risk of tumor development^7^. Additionally, disruptions of the circadian clock can also lead to the development of tumors by affecting the cellular metabolism^8^, by changing the tumor's immune microenvironment^9^, and by interfering with the secretion of melatonin^10^, ^11^. However, current research on the diurnal patterns of symptoms in tumor patients and their molecular mechanisms remains insufficient, which limits clinical insights into diurnal symptom fluctuations and thus is a potential barrier to achieving optimal symptom management and therapeutic outcomes. Therefore, this article aims to systematically analyze the interplay between circadian regulatory networks and tumorigenesis, with a specific emphasis on symptoms following a pattern of “mild in the morning, stable by day, worsening in the evening, and severe at night” and the underlying molecular mechanisms. The objective is to establish a theoretical framework for understanding the chronobiological characteristics of tumor-related symptoms, to develop personalized therapeutic strategies aligned with circadian rhythms, and to optimize clinical treatments.

## The central role of circadian clock genes in the development and progression of tumors

**Fig 1:**
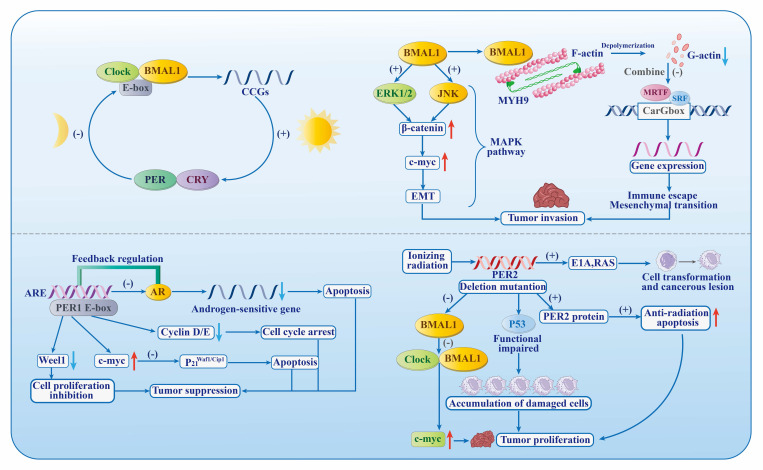
The working mechanism of BMAL1 and PER1 / 2 in normal cells and tumors.

**Fig 2:**
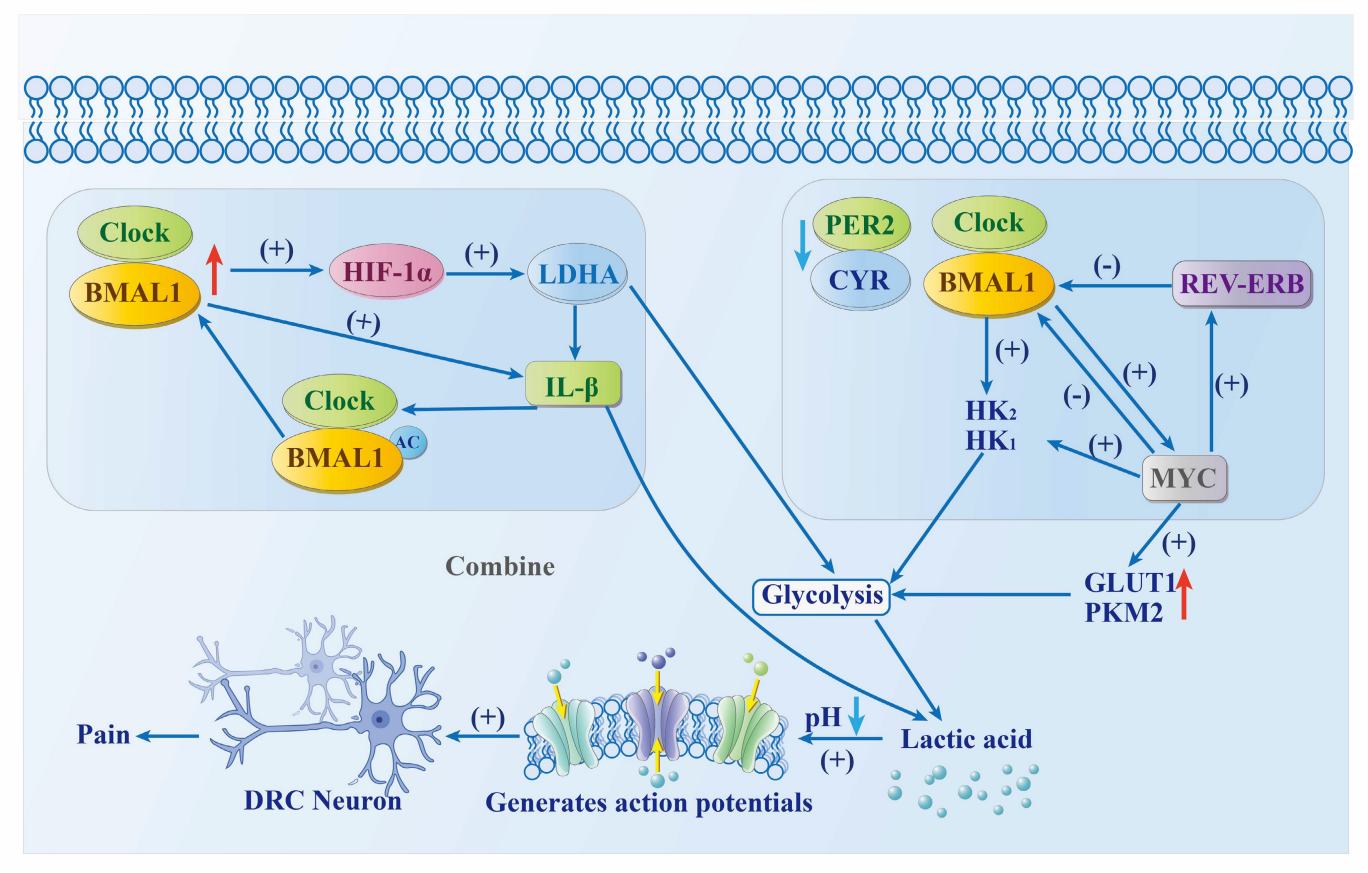
Tumor metabolic reprogramming network.

Circadian clock gene sequences exhibit high levels of evolutionary conservation across taxa and are integral to the transcriptional regulation of circadian rhythms and associated behavioral patterns^12^. The core circadian network primarily consists of core oscillators (such as CLOCK, BMAL1), negative feedback regulators (such as the PER1/2/3 family of period proteins and the CRY1/2 family of cryptochrome proteins), and key regulatory factors (such as the nuclear receptor REV-ERBα, RORα, kinase CK1δ/ε, transcription factor NPAS2, and the Tim protein). These circadian clock genes form a precise network that directly or indirectly regulates a wide range of cellular physiological activities, including circadian rhythms, energy metabolism, autophagy processes, DNA damage repair, protein homeostasis, and cellular secretion. When circadian rhythms are disrupted, these cellular processes are impaired, thereby creating a microenvironment that promotes tumorigenesis, such as metabolic reprogramming, redox homeostasis imbalance, and the formation of chronic inflammation. It is noteworthy that the expression of various circadian clock proteins and secretory factors with pro- or anti-carcinogenic activities is directly regulated by the circadian gene network^13^. To better understand the molecular mechanism involved, Fig.1 and Fig.2 was drawn using Adobe Illustrator 2019.

### The dual role of the BMAL1 gene in tumor development

BMAL1 (Brain and muscle ARNT-like 1) is one of the core transcription factors regulating the circadian rhythm. It forms a heterodimer with the CLOCK protein, constituting the core of the circadian clock's transcription activator complex. As shown in Fig.1, during a normal circadian cycle, the CLOCK-BMAL1 complex binds to E-box sequences in the promoters of target genes during daytime. This drives the transcription of downstream clock-controlled genes (CCGs), including the PER (period) and CRY (cryptochrome) gene families. As PER and CRY proteins accumulate in the cytoplasm and form complexes, they gradually move into the nucleus, where they inhibit the transcriptional activity of CLOCK-BMAL1 during the night^14^. Subsequently, PER and CRY proteins are phosphorylated and degraded via the ubiquitin-proteasome pathway, which reduces their inhibition of CLOCK-BMAL1, thereby restoring its transcriptional function and initiating the next circadian cycle^15^. This very precise feedback loop is the foundation for maintaining the circadian rhythm in organisms and, through its regulation of CCGs, indirectly influences a wide range of physiological processes, such as metabolism, cell cycle, DNA repair, and immune response^16^.

#### Tumor heterogeneity in BMAL1 rhythmic expression

In normal tissues, the transcriptional activity of BMAL1 exhibits circadian fluctuations, typically peaking at night^17^. However, in tumor tissues, the expression rhythm of BMAL1 often exhibits abnormalities. These are: (1) Weakened or absent rhythmicity. In certain tumors such as hepatocellular carcinoma, prostate cancer, and malignant pleural mesothelioma, the rhythmic expression of the BMAL1 gene is significantly weakened or nonexistent^18^-^20^. (2) Altered patterns of rhythmicity. In some tumors or cell lines, BMAL1 still undergoes circadian fluctuations, but they might follow new patterns. For example: In an experiment on mice with lung adenocarcinoma, the overall circadian rhythm remained unchanged, and the transcription of BMAL1 was similar to that in normal tissue^21^. However, in breast cancer cells, the transcription cycle of BMAL1 was prolonged, and its peak expression occurred later, often between 16 and 20 hours (rather than during the night, as would be usual)^22^.

#### The carcinogenic effect of BMAL1

As shown in Fig.1, elevated levels of BMAL1 expression may exert a pro-carcinogenic influence within particular tumor microenvironments by: (1) Promoting immune escape and drug resistance. Highly expressed BMAL1 can bind to the tumor suppressor MYH9, enhancing MRTF-SRF activity and AP-1 transcriptional activity. This induces changes in the cell state of the tumor, which renders it more resistant to immunotherapy and promotes tumor progression^23^. (2) Activating the MAPK/c-Myc pathway. Elevated levels of BMAL1 expression stimulate the MAPK signaling cascade, facilitating the translocation of β-catenin to the nucleus, which in turn enhances the transcription of the oncogenic factor c-Myc, thereby promoting tumor cell proliferation and metastatic progression^24^.

The aforementioned BMAL1-driven carcinogenic processes (such as cell proliferation, invasion, and proto-oncogene expression) may be more active during the night when BMAL1 expression is high (or during specific time periods when its peak phase is delayed). This provides a potential molecular basis for explaining why symptoms in tumor patients (such as pain and discomfort) often worsen at night, just as the previously mentioned expression in traditional Chinese medicine says. Therefore, understanding the expression patterns and functions of BMAL1 in specific tumors helps elucidate the molecular mechanisms underlying the circadian fluctuations of symptoms. This, in turn, is of great significance in chronotherapeutic settings.

#### MAL1 as tumor suppressor

BMAL1 also exhibits significant tumor-suppressive effects in some tumors and physiological contexts. It does so primarily via: (1) Regulation of the tumor microenvironment (TME) and fibrosis. The deletion of BMAL1 genes leads to lower levels of plasminogen activator inhibitor-1 (PAI-1) in the tumor microenvironment, thereby reducing the inhibition of tissue-type plasminogen activator (tPA) and urokinase-type plasminogen activator (uPA). Elevated levels of tPA/uPA catalyze the activation of plasmin, which further promotes the activation of transforming growth factor-β (TGF-β). Activated TGF-β drives pathological fibrosis in tumor tissues, creating a microenvironment conducive to tumor growth and metastasis^25^. Conversely, normal BMAL1 function helps inhibit this pro-fibrotic, pro-metastatic pathway. (2) Weakening capacity for DNA damage repair. Increased BMAL1 expression inhibits the expression of key enzymes involved in DNA repair (such as POLD1, POLD2, and LIG1) and genes related to base excision repair (such as NTHL1, XRCC1, and SMUG1). This reduces the ability of tumor cells to respond to DNA damage (such as double-strand breaks), increases genomic instability, and thereby inhibits tumor cell proliferation and survival^26^. (3) Activation of P53-dependent apoptosis and cell cycle arrest. BMAL1 can directly bind to and activate the promoter of the tumor suppressor gene P53, promoting the phosphorylation (e.g., at Ser15) and stability of the P53 protein. Activated P53 directly induces tumor cell apoptosis. On the other hand, p53 upregulates the expression of its key downstream target gene p21 while downregulating the cell cycle protein cyclin B1. As a potent cyclin-dependent kinase inhibitor (CDKI), p21, in conjunction with reduced cyclin B1, causes cell cycle arrest at the G2/M phase, preventing the abnormal proliferation of damaged cells^27^. Therefore, the absence of BMAL1 leads to a decrease in activated P53 levels, weakening apoptotic capacity and cell cycle checkpoint function. This increases the potential of survival and proliferation of tumor cells and promotes tumor development.

In summary, BMAL1 plays a complex and context-dependent dual role in tumorigenesis and progression. It can promote tumor advancement under certain conditions (such as specific tumor types, expression levels, or phases) by facilitating immune evasion, epithelial-mesenchymal transition (EMT), proliferation, and metastasis, while under other conditions it can inhibit tumor development by maintaining tumor microenvironment (TME) homeostasis, impairing DNA repair, and activating p53 pathways. This functional contradiction suggests that its effects are highly dependent on tissue type, tumor stage, microenvironmental signals, as well as its own expression levels and circadian phase. Given the complexity and context-specificity of BMAL1 functions, its precise mechanistic contribution to circadian phenomena observed in tumor patient symptoms (such as pain and fatigue undergoing a pattern of “mild in the morning, stable by day, worsening in the evening, and severe at night”) remains to be further elucidated. Future research should aim to clarify the circadian patterns of BMAL1 expression and activity across different tumor types and their correlation with symptom severity over time. It should also be further analyzed to which extent pro- and antitumor pathways are activated during periods of high (night) or low (day) BMAL1 activity and what their net effects on symptoms are. Furthermore, chronotherapy strategies should be explored that are based on the rhythm of BMAL1 to suppress its pro-tumor activity while preserving or enhancing its antitumor functions, thereby optimizing symptom management and anti-cancer efficacy.

### PER genes: key tumor suppressors and regulators of the circadian rhythm

The PER genes (Period genes, including PER1, PER2, and PER3) are core members of the circadian clock's negative regulatory feedback loop. In normal circadian rhythms, PER proteins form complexes with CRY proteins, which are transported into the nucleus to inhibit the activity of the CLOCK-BMAL1 transcription activation complex, thereby regulating the expression of downstream clock control genes (CCGs)^16^. PER genes not only regulate circadian oscillations but also serve as critical tumor suppressors by modulating cell cycle checkpoints, controlling cellular proliferation, and inducing apoptosis, through interactions with other circadian clock regulators and pathways involved in oncogenesis^28^.

#### The PER gene as a tumor suppressor: evidence and mechanisms

Numerous studies have demonstrated that PER genes (particularly PER1 and PER2) can act as tumor suppressors. The inactivation of its function as a tumor suppressor (e.g., through gene mutations, promoter hypermethylation, or downregulation of expression) is closely associated with the onset and progression of many tumors. The tumor-suppressing mechanisms involve multiple stages of the cell cycle, as shown in Fig.1: (1) Regulation of cyclin expression and cell cycle progression. Downregulation of PER1 expression leads to increased expression of cyclin D and cyclin E. It also delays peak tumor growth, heightens the amplitude of peak and trough tumor growth, and promotes tumor cell division and proliferation^29^. Conversely, high PER1 expression increases cell arrest in the G2/M phase, reduces the proportion of cells in the S phase, and induces apoptosis, thereby effectively inhibiting tumor cell division and growth^30^. (2) Influence of key oncogene/tumor suppressor gene pathways. Deletion of the PER2 gene inhibits BMAL1 expression via the PER2-BMAL1-c-Myc axis, thereby reducing the levels of functional BMAL1/CLOCK heterodimers. This leads to overexpression of the downstream target gene oncogene c-Myc, causing genomic DNA damage and impairing the function of the tumor suppressor protein P53, ultimately promoting tumor cell proliferation and development^31^. (3) The PER1-androgen receptor (AR) axis. In prostate cancer, PER1 inhibits the transactivation of the androgen receptor (AR), reducing the expression of downstream androgen-sensitive genes, significantly inhibiting tumor growth and inducing cancer cell apoptosis. (4) Weakening of DNA damage response and apoptosis sensitivity. PER2 gene mutations weaken cellular sensitivity to DNA damage-induced apoptosis. For example, PER2 mutant cells exhibit enhanced resistance to apoptosis induced by genotoxic stimuli such as X-rays^32^. Additionally, PER2 mutations can promote cell transformation and tumor formation mediated by oncogenes (e.g., E1A and RAS), increasing the risk of carcinogenesis^32^.

#### The relationship between symptoms and the PER rhythm

The transcription and protein expression of the PER gene exhibit a distinct circadian rhythm, typically peaking during the daytime (light period/active phase) and reaching a low point during the nighttime (dark period/resting phase). As an important tumor suppressor, PER protein's low expression levels during the night may weaken the protective effects of the aforementioned antitumor mechanisms (such as cell cycle arrest, apoptosis induction, oncogene suppression, and DNA damage response). This weakened “protective umbrella” during the night may lead to relatively enhanced tumor cell proliferation, invasion, or inflammatory activity, providing another important molecular perspective to explain why symptoms in tumor patients (such as pain and discomfort) worsen at night. Therefore, restoring or enhancing the rhythmic expression of the PER gene may become one of the strategies to improve night-time symptoms.

## The circadian clock as a regulator of tumor metabolism, immunity and endocrine function

### Circadian disruption and metabolic reprogramming of tumor cells

In tumor cells, the circadian clock's rhythm is disrupted. The interaction between an abnormal circadian rhythm and the metabolism supplies tumor cells with large amounts of metabolites and energy to support their proliferation, survival, invasion, and metastasis. Tumor cells enhance cellular metabolic adaptability by regulating activities such as glycolysis, glutamine degradation, protein, lipid, nucleic acid synthesis, and mitochondrial function^33^. Revealing the relationship between the circadian clock and tumor cell metabolism has significant implications for the development of drugs targeting tumor metabolism.

#### The circadian regulation hub of normal metabolism

The metabolic processes of normal cells are regulated by the circadian rhythm, with signal transduction mediated by REV-ERBα. Two mechanisms illustrate how this mediation occurs. (1) CLOCK/BMAL1-PPARα-REV-ERBα axis. The CLOCK-BMAL1 heterodimer transactivates PPARα, a key regulator of fatty acid metabolism, through the receptor response element PPRE, thereby upregulating the expression of REV-ERBα in tissue cells^34^. (2) The feedback loop of REV-ERBα. REV-ERBα can bind to the RORE/RevRE sites on the promoters of its target genes, regulating the expression of circadian rhythm factors such as BMAL1 and participating in the circadian rhythm control of processes such as bile acid metabolism. At the same time, it influences lipid metabolism by downregulating the expression of hepatic apolipoprotein C-III and promoting the differentiation of preadipocytes into mature adipocytes^35^.

#### Two main pathways of tumor metabolic reprogramming

The circadian clock regulates the glycolytic process by modulating the expression of MYC and hypoxia-inducible factor 1α (HIF-1α) in tumor cells. This is achieved through two mechanisms, as shown in Fig.2. (1) Tumor growth feedback autoregulatory loop. Hypoxia-inducible factor-1 alpha (HIF-1α) functions as a key transcriptional regulator of glycolytic metabolism, with its activity directly modulated by the circadian clock component CLOCK. The interaction between HIF-1α and BMAL1 amplifies its transcriptional activity, resulting in upregulation of lactate dehydrogenase A (LDHA) expression, which in turn enhances glycolytic flux and lactate biosynthesis. The secretion of lactate and interleukin-1β (IL-1β) regulates BMAL1 deacetylation, which promotes CLOCK/BMAL1-mediated expression of LDHA and IL-1β. This further enhances glycolysis^36^. (2) MYC central pathway. The absence or mutation of BMAL1, PER2, and cryptochrome 2 (CRY2) expression leads to increased MYC levels. This promotes REV-ERBα expression, which in turn inhibits BMAL1 transcription and promotes hexokinase 2 (HK2) expression, thereby promoting glycolysis. The expression of HK2, glucose transporter type 1 (GLUT1), pyruvate kinase M2 (PKM2), and lactate dehydrogenase A (LDHA) can be directly induced by MYC to enhance glycolysis. The expression of CLOCK and BMAL1 can be inhibited by MYC, which promotes glycolysis and lactate production by upregulating the protein levels of hexokinase 1 (HK1) and LDHA^36^.

#### The relationship between metabolic disorders and symptoms

Tumor cells are more active at night than during the day^37^. This increase in energy demand promotes glycolysis, leading to a large accumulation of lactic acid in the cytoplasm. This, in turn, results in a reduction of acidity in the pH scale and activates acid-sensitive ion channels (ASICs), exacerbating pain and discomfort in tumor patients at night^38^ and leading to symptoms “worsening in the evening, and (being) severe at night.” Therefore, weakening the metabolic process of cells at night helps to inhibit tumor development and improve night-time symptoms in tumor patients.

### The circadian rhythm as a regulator of tumor immunity

The immune system is the most effective weapon against tumors and cancers, which is why tumor immunotherapy has been so widely used. Tumor immune surveillance is an important tumor suppressor mechanism that protects the body from tumor invasion. Its intensity varies with the circadian rhythm. Some studies have shown that both innate and adaptive immunity exhibit distinct circadian rhythmicity. The circadian clock can enhance adaptive immunity and regulate immune function by controlling the transport of inflammatory monocytes and macrophage-mediated inflammatory responses^39^.

#### Circadian fluctuations in immune surveillance

First, there is the rhythmicity of immune cell transport. Lymph nodes are one of the body's most important immune structures. They effectively capture lymphoid antigens, antigen-presenting cells, and lymphocytes, while also playing a regulatory role in the body's adaptive immune response^40^. During nocturnal hours, leukocyte circulation elevates, with dendritic cells and lymphocytes exhibiting maximal homing activity to lymphoid tissues^41^. This enhances antigen processing and presentation, facilitates the recognition of non-self antigens, and strengthens immune defense mechanisms. As a result, the adaptive immune response reaches its peak during early morning hours. Second, there are the pro-tumor effects of circadian rhythm disruption. Alterations in circadian rhythm factors in tumor cells, such as the absence or mutation of PER2 and BMAL1, can induce the expression and activation of the proto-oncogene c-Myc and enhance metabolic processes like glycolysis, further promoting tumor proliferation and development^42^. When the circadian rhythm is completely disrupted (SCN damage), tumor growth can accelerate by a factor of 2 to 3^43^.

#### Time-dependent mechanisms of immune clearance

First, there is the circadian rhythm of CD8⁺T cells. CD8^+^T lymphocytes and dendritic cells (DCs) both exhibit circadian-regulated antitumor immune functions, with their populations peaking in the afternoon and reaching nadirs at night. Consequently, tumor inoculation during the night results in accelerated tumor growth and larger tumor volumes. Dendritic cells undergo circadian trafficking to tumor-draining lymph nodes, where the circadian expression of the co-stimulatory molecule CD80 modulates the circadian response of CD8^+^T cells, thereby promoting their activation and influencing tumor progression^44^. Then there are the circadian dynamics of tumor-infiltrating lymphocytes (TILs). The temporal dynamics of TILs in melanoma murine models exhibit significant circadian variation, with peak infiltration observed nocturnally, correlating with maximal antitumor activity. T cells show marked enrichment in leukocyte adhesion and T cell activation signaling pathways, which are positively associated with patient survival outcomes. This circadian pattern has been validated in human melanoma patients, where CD4^+^ and CD8^+^T cell populations also display diurnal fluctuations, reaching their zenith in the afternoon and decreasing during the night^45^. Finally, there is rhythmic inhibition of PD-1. The immune checkpoint protein PD-1 is an important immunosuppressive molecule that downregulates immune cell proliferation, cytokine secretion, and cytotoxicity, thereby preventing effector T cells from killing cancer cells^46^. The expression pattern of the immune checkpoint gene PDCD1, which encodes PD-1, in CD8^+^T lymphocytes demonstrates a circadian oscillation, with peak levels observed in the morning and nadir levels at night. Therefore, administering anti-PD-1 immunotherapy during morning hours may optimize immune activation, thereby eliciting the most potent cytotoxic response against neoplastic cells^45^.

#### Imbalance of macrophage polarization

Disruptions of the circadian rhythm induce fluctuations in the quantity and phenotypic composition of macrophages over a 24-hour cycle. Activated macrophages are typically classified into pro-inflammatory M1 macrophages and anti-inflammatory M2 macrophages. M1 macrophages exhibit anti-tumor properties by secreting pro-inflammatory cytokines (such as IL-12 and TNF-α) and employing direct cytotoxic mechanisms (reactive oxygen species and reactive nitrogen species) to inhibit tumor cell proliferation and survival. Conversely, M2 macrophages promote tumor progression through the secretion of anti-inflammatory factors (such as IL-10 and TGF-β) and by facilitating tumor angiogenesis, thereby creating an immunosuppressive microenvironment that supports tumor growth and aids in immune evasion^47^. In healthy subjects, M1 and M2 macrophages display circadian oscillations, with M1 macrophage levels markedly reduced during daytime compared to nighttime, whereas M2 macrophage levels are elevated during the day. The M1/M2 polarization ratio peaks nocturnally. Chronic jet lag (CJL) markedly diminishes nocturnal M1 macrophage counts and abolishes the circadian variation in M2 macrophage levels. Consequently, the M1/M2 ratio declines significantly, resulting in increased immune tolerance, which may facilitate tumor immune evasion and promote tumor initiation, growth, and progression^48^.

#### The relationship between immune rhythms and symptoms

At night, the number of CD8^+^ T cells reaches a low point, leading to weakened immune surveillance. Meanwhile, the polarization state of macrophages undergoes alterations, with a decrease in the M1/M2 ratio, suggesting an enhancement of the body's immune tolerance. A persistently low M1/M2 ratio indicates a poor prognosis for tumors. Under this immunosuppressive microenvironment, the immune evasion mechanisms of tumor cells are amplified, leading to a significant increase in their proliferative activity. Changes in immune status during the night are often accompanied by intensified local or systemic inflammatory responses^47^, potentially exacerbating clinical symptoms such as pain and discomfort, which are more notably perceived during nighttime. Notably, the transcriptional expression of PD-1 also reaches its nadir at night, indicating that the inhibitory effect of the PD-1/PD-L1 immune checkpoint pathway on downstream immune responses is relatively diminished. Based on this circadian rhythm, the night may serve as a potential "immune therapeutic window." Administering immune checkpoint inhibitors targeting the PD-1/PD-L1 pathway during this period could theoretically enhance anti-tumor immunity due to a reduction in endogenous suppression, thereby improving therapeutic efficacy.

### Endocrine circadian rhythms drive tumor metastasis

In recent years, the interplay between the endocrine system and tumors has attracted significant scholarly interest. The endocrine system orchestrates critical physiological processes via the biosynthesis and secretion of hormones. Dysregulation within this system can contribute to neoplastic transformation, while tumors can reciprocally modulate endocrine function by altering hormone secretion profiles. For example, hyperestrogenism has been associated with an increased risk of mammary carcinogenesis, potentially through the upregulation of progesterone receptor expression and enhancement of progesterone-mediated signaling pathways, thereby elevating carcinogenic susceptibility^49^. Similarly, pulmonary malignancies may stimulate thyroid hormone secretion, leading to abnormal elevations in free thyroxine (FT4) levels during advanced disease stages^50^. This review emphasizes the influence of circadian fluctuations in melatonin secretion on tumorigenesis. Melatonin is an indole compound which, under physiological conditions, is primarily synthesized and secreted by the pineal gland and regulated by the suprachiasmatic nucleus (SCN). In healthy individuals, melatonin synthesis peaks between midnight and 3 a.m., with peak levels ranging from 100 to 200 pg/mL. Following this, melatonin synthesis gradually decreases, and levels remain consistently low during the day (10-30 pg/mL)^51^.

#### The dual role of melatonin

Synthetic melatonin can exert a reverse effect on the SCN, thereby promoting sleep and synchronizing circadian rhythms as well as sleep-wake cycles through time-dependent effects and fluctuating concentrations^52^. Melatonin levels in tumor patients are significantly lower than in healthy individuals^53^-^55^, particularly in estrogen receptor-positive (ER^+^) breast cancer patients, where the absence of a nocturnal peak may predict the onset of breast cancer^56^, ^57^. This may be related to the weakened antiproliferative effect of melatonin, which inhibits the binding of the oestradiol-oestrogen receptor complex to the oestrogen response element and blocks ER gene transcription^58^, ^59^. Additional evidence suggests that in early-stage breast tumors, melatonin concentrations increase in both blood and the pineal gland, but decrease as the tumor grows further^60^. This may be related to the role melatonin might be playing at different stages of tumor progression, including initial activation, inhibition of tumor growth, and reactivation of tumor cell metastasis. All this adds to the complexity of assessing the role of melatonin in tumor development^61^.

#### The nocturnal diffusion mechanism of CTCs

The hallmark of tumor progression and metastasis is the dissemination of tumor cells, a process facilitated by circulating tumor cells (CTCs). Clinical evidence indicates that CTC counts in multiple myeloma and breast cancer patients reach peak levels during nighttime (resting/sleep periods), as well as exhibiting higher proliferation rates and metastatic potential^37^, ^62^. Animal experiments also confirm this conclusion, as melatonin can significantly induce the production of individual CTCs, CTC clusters, and CTC-WBC clusters in tumor-bearing mice, which accelerates tumor metastasis. This effect can be attenuated by melatonin receptor antagonists^37^.

#### The relationship between melatonin and symptoms

The synthesis and secretion of melatonin exhibit a distinct circadian rhythm, typically peaking during the night (dark phase/resting phase) and gradually decreasing to consistently lower levels during the day (light phase/active phase)^51^. Therefore, the abundant secretion of melatonin at night induces the production and release of CTCs, enabling tumors to metastasize and disseminate throughout the body. This process involves the rapid and massive proliferation of tumor cells to form secondary metastatic lesions. It also involves exacerbated night-time pain and systemic symptoms in patients brought about by the mechanical compression or release of inflammatory cytokines. However, as previously mentioned, melatonin exhibits complex roles in tumor progression, with its peak deficiency potentially increasing the risk of breast cancer development. This outcome may be associated with tumor histology, methodological variations across studies, and the varying functions of melatonin at different stages of tumorigenesis. Consequently, the clinical application of melatonin in tumor treatments should be integrated with individualized assessment. Future research through high-quality clinical trials will be essential to clarify its precise role across various cancer types and to optimize therapeutic strategies.

## Tumor progression exacerbates pain and sleep disturbances

The progression of tumors is closely associated with a significant exacerbation of patient pain and the onset of sleep disturbances. Its pathophysiological mechanisms are profoundly linked to widespread disruptions in the circadian system. As previously described, cancer patients often exhibit dysregulation in core circadian clock genes such as BMAL1 and PER1/2, accompanied by abnormalities in rhythmic activity within metabolic, immune, and endocrine systems. These alterations collectively create a microenvironment conducive to tumor progression during nighttime and interact with pain and sleep disturbances through mechanisms involving the following aspects: (1) Direct infiltration of the sleep-wake regulatory centers by the tumor: Tumor proliferation or metastasis can directly invade critical hypothalamic structures involved in sleep-wake regulation, disrupting their normal function. This process may impair the synthesis and secretion of key circadian hormones such as melatonin, thereby compromising sleep architecture and circadian stability, resulting in sleep fragmentation and rhythm disturbances^63^. (2) Neurotropic invasion and neuropathic pain: tumors can infiltrate within or surrounding nerve bundles via neurotropic pathways, leading to mechanical compression or chemical damage of neural structures, thereby inducing neuropathic pain^64^, ^65^. This type of pain is often associated with sympathetic nervous system hyperactivity, resulting in physiological responses such as tachycardia and hypertension, which consequently lead to sleep initiation difficulties, sleep maintenance disturbances, or frequent nocturnal awakenings. (3) The interaction between tumor and immune responses and the release of pro-inflammatory cytokines: the crosstalk between tumor cells and the immune microenvironment can lead to dysregulated secretion of various pro-inflammatory cytokines (such as TNF-α, TGF-β, IL-10, IL-1β, IL-6, etc.)^66^, ^67^. These cytokines can traverse the blood-brain barrier or influence the central nervous system via neuro-immune pathways, disrupting the sleep regulation circuits, particularly affecting the stability of REM sleep, and clinically presenting as complex sleep disorders such as insomnia, hypersomnia, or nocturnal arousals. (4) Tumor metabolic reprogramming and nociceptive sensitization: The widespread metabolic alterations in cancer cells, particularly the Warburg effect leading to enhanced glycolysis, result in local lactic acid accumulation. The accumulated lactic acid can activate acid-sensitive ion channels on peripheral nerve endings, subsequently stimulating nociceptive neurons in the dorsal root ganglion (DRG), thereby inducing or exacerbating pain perception^38^. Pain itself serves as a potent arousal stimulus, potentially further disrupting sleep continuity. (5) Disruption of endocrine circadian rhythms and melatonin secretion deficiency: cancer patients frequently exhibit circadian dysregulation of the endocrine axis, notably characterized by a significant reduction in melatonin secretion levels^53^-^55^. Melatonin, as a key endogenous signaling molecule regulating the circadian sleep-wake cycle, whose deficiency can directly result in circadian rhythm disorders, exacerbating issues such as insomnia and premature awakening.

In summary, these mechanisms are not isolated events but are intricately interconnected and mutually amplifying within the tumor progression process, collectively forming the fundamental pathophysiological basis for the exacerbation of nocturnal symptom burden. Therefore, a comprehensive understanding of the etiopathogenesis of tumor-associated pain and sleep disturbances from a chronobiological perspective holds significant clinical implications for the development of chronotherapy strategies and the improvement of patients' quality of life.

## Treatment and transformation

Chronotherapy primarily involves three categories: circadian clock training therapy, timed pharmacological administration, and targeted chronotherapy. Its fundamental goal is to maximize anti-tumor efficacy and minimize treatment-related adverse effects by optimizing the timing of interventions based on the circadian rhythm^68^. Clock training therapy aims to maintain or restore the body's natural diurnal rhythm, thereby reducing tumor interference with physiological functions. Light therapy is a key modality within this approach. Research has demonstrated that exposure to light is a primary regulator of circadian rhythms and melatonin secretion, capable of inducing phase shifting or resetting the biological clock^69^. Light therapy not only stabilizes circadian rhythms but also enhances sleep efficiency, prolongs sleep duration, and reduces sleep latency^70^. Clinical studies involving breast and prostate cancer patients have reported improvements in symptoms such as fatigue, sleep disturbances, depression, and anxiety^71^-^74^. Although red light has been shown to promote sleep and timed light exposure can improve therapeutic outcomes^72^, morning exposure to bright white light is more effective than dim red light and can alleviate daytime activity impairments in female chemotherapy patients^73^.

Timed pharmacologic administration maximizes therapeutic efficacy by aligning dosing schedules with endogenous biological rhythms, such as circadian variations in pharmacodynamics, toxicity profiles, and pharmacokinetics^75^. Meta-analytical data reveal that immune checkpoint inhibitor (ICI) efficacy is temporally dependent, with morning dosing eliciting a more potent humoral immune response, consistent with circadian patterns of immune cell activity and trafficking^76^. This correlates with peak T lymphocyte counts and PD-1 receptor expression in the early hours, whereby treatment during this period optimizes effector T cell activation and proliferation, thereby enhancing anti-tumor immunity and disease control^77^. PD-L1 ligand expression also exhibits circadian oscillations, reaching maximal levels during initial activity; thus, administering anti-PD-L1 agents within this window can potentiate immune cell infiltration and anti-neoplastic activity, effectively inhibiting tumor progression^9^. Multiple oncological studies across diverse malignancies consistently demonstrate that morning immunotherapy administration results in improved clinical outcomes compared to afternoon or nocturnal dosing, with a corresponding increase in median patient survival^78^, ^79^. These findings highlight the pivotal influence of circadian biology in modulating immune-based chemotherapeutic interventions.

Targeted chronotherapeutic strategies predominantly utilize small molecule modulators to regulate circadian rhythm-associated proteins, thereby avoiding the complexities inherent in direct genetic modification. For example, carbazole derivatives like KL001 interact with the flavin adenine dinucleotide (FAD) binding domain to stabilize cryptochrome (CRY) proteins, leading to downregulation of stemness-associated gene expression in glioma stem cells and subsequent inhibition of tumor growth^80^. ROR-γ acts as a transcriptional activator of the androgen receptor (AR) gene by binding to RORE elements, which promotes the progression of castration-resistant prostate cancer. Small molecule antagonists can disrupt this interaction, decreasing AR levels and delaying disease progression^81^. Furthermore, CKIα inhibitors activate the p53 tumor suppressor pathway and target transcriptional kinases such as CDK7 and CDK9, impairing oncogenic transcriptional elongation and synergistically inducing apoptosis, thereby exerting anti-leukemic effects^82^.

Levels of melatonin, a pivotal regulator of circadian physiology, are intricately correlated with oncogenic progression. Bioinformatics research has demonstrated that melatonin-modulated gene expression is markedly dysregulated across diverse malignancies, a pattern associated with somatic mutations and exhibiting tumor-specificity^83^. The aberrant expression of these regulatory genes facilitates the activation of oncogenic signaling pathways and may serve as biomarkers for patient prognosis. Furthermore, pharmacogenomic analyses have identified multiple anti-cancer agents which might be able to target this regulatory network^84^. Consequently, a comprehensive exploration of melatonin's regulatory mechanisms will advance our understanding of tumor pathogenesis and inform the development of innovative therapeutic interventions.

In summary, chronotherapy aims to harness endogenous circadian rhythms to dynamically optimize the timing, dosage, and regimen of anticancer pharmacotherapy. This approach seeks to intervene within a temporal window when tumor cells exhibit maximum sensitivity to treatment and normal tissue toxicity tolerance is highest, thereby maximizing therapeutic efficacy and minimizing adverse effects. Given the close association between melatonin-modulated gene and tumor progression, targeted modulation of melatonin signaling axis has emerged as a promising innovative strategy in tumor treatments. Future research priorities may focus on developing highly selective melatonin receptor agonists/antagonists; exploring temporally optimized combination therapies integrating melatonin with conventional chemotherapy, radiotherapy, and immune checkpoint inhibitors; and utilizing systems biology coupled with artificial intelligence models to integrate multi-omics data for predicting individual circadian phase, thereby facilitating the clinical translation of personalized chronotherapy.

## Conclusion

The circadian clock influences the pathogenesis and progression of malignant tumors through several regulatory pathways. These are: (1) Circadian oscillations of clock genes (BMAL1, PER1/2) influencing cell cycle regulation and signal transduction pathways within the tumor microenvironment, thereby facilitating tumor proliferation and metastasis at night. (2) Metabolic reprogramming of neoplastic cells enhancing glycolytic flux to meet energy demands for nocturnal cellular proliferation. (3) A reduction in immune effector cell populations during the night, impairing immune surveillance and enabling tumor immune escape. (4) Elevated nocturnal secretion of melatonin, which promotes the formation of circulating tumor cells and accelerates metastatic dissemination. The heightened tumor cell proliferation and dissemination at nighttime exacerbate clinical symptoms such as pain and sleep disturbances by way of direct tissue invasion, neural infiltration, induction of pro-inflammatory cytokines, activation of dorsal root ganglion neurons, and suppression of melatonin synthesis. In clinical oncology, chronotherapeutic approaches—including phototherapy, timed pharmacotherapy, and circadian rhythm modulation—can optimize anti-tumor efficacy, while monitoring melatonin levels may serve as a prognostic biomarker and facilitate the development of therapeutic interventions that are based on circadian rhythms. While valuable insights have been obtained, some limitations remain. This study focuses on specific types of circadian clock genes, metabolic signaling pathways, immune cell subsets, and melatonin-related biomarkers. Future studies will aim to expand circadian gene profiling, conduct detailed analyses of the regulatory networks controlling metabolic pathways, and systematically explore additional immune cell phenotypes and hormonal effects. The goal is to develop a more comprehensive and accurate model of circadian regulation and physiological homeostasis.
